# Assessment of addition of biochar to filtering mixtures for potential water pollutant removal

**DOI:** 10.1007/s11356-017-0650-6

**Published:** 2017-11-07

**Authors:** Lea Piscitelli, Pierre-Adrien Rivier, Donato Mondelli, Teodoro Miano, Erik J. Joner

**Affiliations:** 1 Mediterranean Agronomic Institute of Bari (CIHEAM), Via Ceglie, 9, Valenzano, 70010 Bari, Italy; 2 Division of Environment and Natural Resources, Norwegian Institute of Bioeconomy Research, Høyskoleveien 7, 1431 Ås, Norway; 3 Department of Food, Plants and Soil Science, University of Bari Aldo Moro, Via G. Amendola 165/a, 70126 Bari, Italy

**Keywords:** Green roof substrates, Filtering mixtures, Biochar, Heavy metals, Phenanthrene, Column experiment

## Abstract

Green roofs are used increasingly to alleviate peaks of water discharge into the sewage systems in urban areas. Surface runoff from roofs contain pollutants from dry and wet deposition, and green roofs offer a possibility to reduce the amounts of pollutants in the water discharged from roofs by degradation and filtering. These pollutants would otherwise enter wastewater treatments plants and ultimately end up in sewage sludge that is spread on agricultural soils. The most common substrates used in green roofs have limited capacity for filtration and sorption. Also, more sustainable alternatives are sought, due to the high carbon footprint of these materials. Biochar is a carbon-rich material produced by pyrolysis of biomass, and several types of biochar have been described as good sorbents and filter materials. Biochar is also a light and carbon negative material, which may fulfill other desired criteria for new green roof substrates. We here report on an experiment where two types of biochar, produced from olive husks at 450 °C or from forest waste at 850 ° C were mixed with volcanic rock or peat, and tested for retention capacity of phenanthrene and six heavy metals in a column experiment with unsaturated gravimetric water flow lasting for 3 weeks. The results suggest that biochar as a component in green roof substrates perform better than traditional materials, concerning retention of the tested pollutants, and that different types of biochar have different properties in this respect.

**Electronic supplementary material:**

The online version of this article (10.1007/s11356-017-0650-6) contains supplementary material, which is available to authorized users.

## Introduction

Climate change is expected to cause increased frequency and intensity of rainfall, resulting in peaks of surface runoff from impermeable surfaces in urban areas. A strategy to mitigate urban flooding problems consists of retarding runoff using green roofs and similar flow-reducing installations. An additional environmental challenge comes from dissolved and particulate pollutants in precipitation and runoff from impermeable surfaces. These end up in sewage treatment plants and are ultimately discharged as sewage sludge into soils where they may transfer to plants and animals and the human food chain (Mench et al. 2009). In order to reduce the amount of contaminants that enter arable soil through this route, filtering and biodegradation in green roofs and other flow-reducing installations may become significant. The use of green roofs is increasing, and they are even considered an efficient and sustainable way to reduce the occurrence of pollutants in the environment (Dierkes et al. 2006). The pollutant removal capacity does however depend on the materials used and their inherent properties. At the same time, new roof materials are sought to reduce material costs and improve their overall environmental footprint. Currently used green roof materials feature, among others, volcanic rock and peat, which are light and inexpensive, but which are either exotic to large parts of the World, resulting in long-distance transport, or considered as non-sustainable due to negative climatic and environmental consequences of excavation and removal (Alexander et al. 2008).

Biochar can be produced by pyrolysis of any type of biomass and is a material characterized by a high porosity and a high-specific surface area. This porosity, along with a variety of both charged and hydrophobic surface micro sites, gives biochar favorable properties both for retaining water and for sorbing both ions and non-polar molecules. Biochar has positive effects on plants and is considered a good soil improver (Ennis et al. 2012). However, some variation for these properties is found between different types of biochar owing to differences of the parent biomass materials and pyrolysis conditions (Schimmelpfennig and Glaser 2011).

Heavy metal (HM) adsorption to biochar involves several types of interactions, including ion exchange, physical adsorption, electrostatic attraction, surface complexation, and precipitation and reduces their bioavailability and phytotoxicity (Park et al. 2011). On the other hand, the binding of organic contaminants to biochar often feature electrostatic interactions, hydrophobic effects, hydrogen bonds, and pore-filling (Ulrich et al. 2015).

Combining peat with biochar can result in a carbon neutral material due the carbon negative nature of biochar and its high resistance to degradation processes. Moreover, the high porosity and the large surface area of biochar can improve peat adsorption by ion exchange and surface complexation. In addition, the capacity of volcanic rock to retain water and remove contaminants can be improved by the addition of biochar, which potentially contributes to the increase of active sites and hydrophilic functional groups on the particle surface of the mixtures (Reddy et al. 2014).

Choice of feedstock and pyrolysis conditions makes it possible to enhance specific physiochemical properties of biochar (Verheijen et al. 2009). Thus, using biochars characterized by different properties regarding adsorption of pollutants could lead to desirable adsorption and filtering capacities. In order to evaluate the contribution of contrasting biochars for removing dissolved pollutants, each of two types of biochar (produced from olive husks or forest waste), were tested in a static batch incubation experiment and then in a dynamic column experiment.

The aim of this study was to assess whether the conventional green roof materials peat and volcanic rock could be improved regarding adsorption for heavy metals and phenanthrene (as a representative for organic contaminants with limited water solubility), when amended with either of two contrasting types of biochar. This was done by comparing key physico-chemical characteristics of the materials and mixtures, by measurements of the capacity to adsorb of the individual materials, and by assessing the filtering capacity of material mixtures during water percolation in a column experiment.

## Materials and methods

### Experimental design

A column experiment with four substrate combinations plus an overlaying sand layer spiked with phenanthrene (Phe) was set up with three replicate columns per treatment and percolated with an aqueous solution of heavy metals during three weekly episodes. Percolating water was recovered and analyzed to determine the capacity of the substrate mixtures to retain environmental pollutants. The four substrate combinations contained either 70% peat or 70% volcanic rock in combination with 30% of either of two types of biochar (*v*/*v*) (30% is the maximum amount permitted for use in roof constructions according to risks of fire).

### Materials

Peat (fibrous and only weakly transformed, limed to pH 6.5) was purchased from Nittedal Torvindustri AS (Arneberg, Norway) and volcanic rock (VR) from Veg Tech (Stockholm, Sweden). Wood biochar (BP), produced from mixed forest waste at 850 °C by slow pyrolysis was purchased from Pyreg GmbH (Dörth, Germany). Olive husk biochar (BO) was produced by slow pyrolysis in an experimental reactor at 450 °C using air-dried olive husks by the Mediterranean Agronomic Institute of Bari (Valenzano, Bari, Italy). Some additional data about BO and BP are reported in Table S1 in supplementary information.

Prior to use, all materials were wet-sieved and the fraction between 1 and 2 mm retained, ensuring homogeneous particle distribution and avoiding particle-bound transport of the added contaminants.

The treatments obtained in this way were named peat/BP, peat/BO, VR/BP, and VR/BO from their constituents.

### Materials characterization

Over-dried materials (105 °C) were characterized with respect to pH (sample to water ratio of 1:2.5, *w*/*v*), Eh, bulk density, and water holding capacity (WHC). The latter involved packing materials in volumetric cylinders with porous bottoms, soaking in water overnight, draining for 24 h on a saturated sandy surface, and measuring the retained water at 10-cm height gravimetrically.

### Static adsorption

In order to verify that metal concentrations to be used in a dynamic system were suitable, a batch adsorption experiment was conducted. Individual materials were thus assessed using aqueous solutions of metal ions and Phe at three different concentrations. Phenanthrene (1.6 mg l^−1^) and 5 mg l^−1^ of each of Cd (CdSO_4_), Cu (CuSO_4_), Cr (K_2_CrO_4_), Ni (NiCl_2_), Pb (PbCl_2_), and Zn (ZnSO_4_) were used undiluted (high loading; [H]) or diluted 1:1 (medium; [M]) or 1:10 (low; [L]). Three grams (d.wt.) of each substrate were placed in 100 ml flasks and incubated in triplicate for 16 h at 23 °C with 60 ml (1,20, w,v) of the three solutions ([H], [M], and [L]) prior to analyzing the remaining unabsorbed pollutants. The extraction of the unabsorbed Phe was made by loading 20 ml of the solutions on Bond Elut Plexa cartridges followed by elution with dichloromethane-ethyl acetate (6,4) and analysis by gas chromatography-mass spectrometer (GC-MS) (Agilent 6890) using a HP-5MSi column (Agilent 19091S-433). The MS detection was carried out in SIM mode with a mass-to-charge ratio of 152.00. For metal analyses the incubated solutions were filtered using Whatman grade 1 paper filters and acidified with HCl before analysis by Induced Coupled Plasma–Optical Emission Spectroscopy (ICP-OES) (ICAP 6300, Thermo Electron, UK).

Control treatments, containing 3 g of each material and 60 ml of water, were included and analyzed to account for metals and phenanthrene in the materials and from secondary pollution (see Table S2).

### Column experiment

A column experiment was performed in order to explore the capacity for removal of pollutants in a dynamic system, comparing the performances of the four mixtures to the two traditional roof materials. Stainless-steel columns (10 cm diam.) with steel mesh bottoms fitted with filter paper to retain particles were filled with either of the four material mixtures to a height of 20 cm (1570 cm^3^), and topped with a 2-cm layer of sand. Before the addition of the sand layer, columns containing the materials/material mixtures were percolated with 300 ml of pure water in order to equilibrate the system and to verify the absence of pollutants in the percolated water. This sand had been spiked with Phe by adding 10 ml of acetone containing 200 mg Phe L^−1^ to each portion of 250 g of sand to reach a final concentration of 8 mg kg^−1^of Phe. The spiked sand layer also ensured a uniform solution flow in the underlying material, while at the same time continuously releasing Phe through elution by the percolating water. A control treatment containing only Phe-spiked sand was included to verify the elution of Phe during percolation with water. At three occasions (day 7, day 14, and day 21), fixed volumes (300 ml) of metal-spiked solution (5 mg l^−1^, same as used in the static experiment “Static adsorption”) prepared for each individual column was pumped onto the sand layer and throughout the substrates using a peristaltic pump with a flow rate at approx. 1 ml min^−1^.

### Statistical analysis

All data were statistically analyzed using one-way analysis of variance (ANOVA) and SPSS software. Means were compared by using the least significant difference test (LSD test) at *p* < 0.05.

## Results and discussion

### Material characteristics

As shown in Table 1, the pH of the two biochars was alkaline, but not significantly different, whereas VR had a less alkaline pH. The commercial peat contained some lime and had a slightly acidic pH. Steiner and Harttung (2014) reported that peat could be mixed with up to 80% of biochar without raising the pH above 7. In our study, both peat-based mixtures gave increased pH compared to pure peat when amended with 30% of biochar. All the analyzed mixtures had a pH in the optimal range for retention of heavy metals, yet not as high as to impede microbial activity and plant growth, or functioning as a filter material. Materials and mixtures proposed for this study had a low bulk density, which both facilitate transportation and handling, reduces the need for enhanced carrying capacity of roof constructions, and reduces the onset of clogging problems (Spychala and Ejewski 2003). Except VR, the materials and mixtures had a high WHC, which would also increase water retention time and provide suitable conditions for plant growth. An increased retention time of a green roof substrate results in both reduced peak roof out-flow and enhanced offset of water reaching the sewage system, and potentially increase pollutant adsorption on the substrate, as increased contact time allows for higher absorption of pollutants (Dalahmeh et al. 2012).

**Table 1:** Characteristics of materials and mixtures. VR volcanic rock, BP biochar from wood, BO biochar from olive husks, WHC water holding capacity

		VR	BP	BO	Peat	VR/BP	VR/BO	Peat/BP	Peat/BO
pH_water_	1:2.5	7.8	9.5	9.7	5.8	8.5	8.7	7.2	7.9
Density	g/cm^3^	0.5	0.2	0.4	0.3	0.3	0.5	0.3	0.4
WHC	%	42	258	73	149	112	54	205	143

### Static retention capacity

**Fig. 1:**
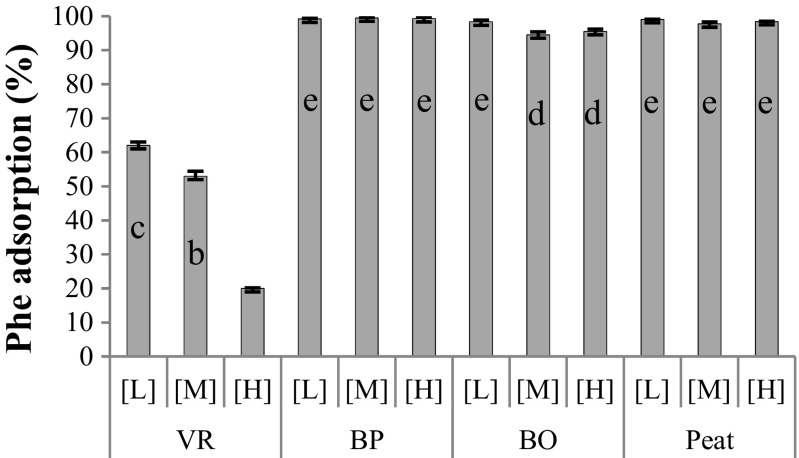
Phenanthrene (Phe) adsorption of individual materials at different concentration (high loading; [H]), diluted 1:1 (medium; [M]), or 1:10 (low; [L]). Different letters above bars indicate significant difference between means (*p* < 0.05, LSD test, *n* = 3)

As shown in Fig. 1, VR had the lowest capacity in terms of Phe adsorption under static conditions and only retained 20–60% of the Phe, the lowest values observed at high Phe loading rates. Phe adsorption for the other materials was high and indicated high affinities for Phe. The Phe absorption efficiency was not significantly different among the materials BP, BO, and Peat.

**Fig. 2:**
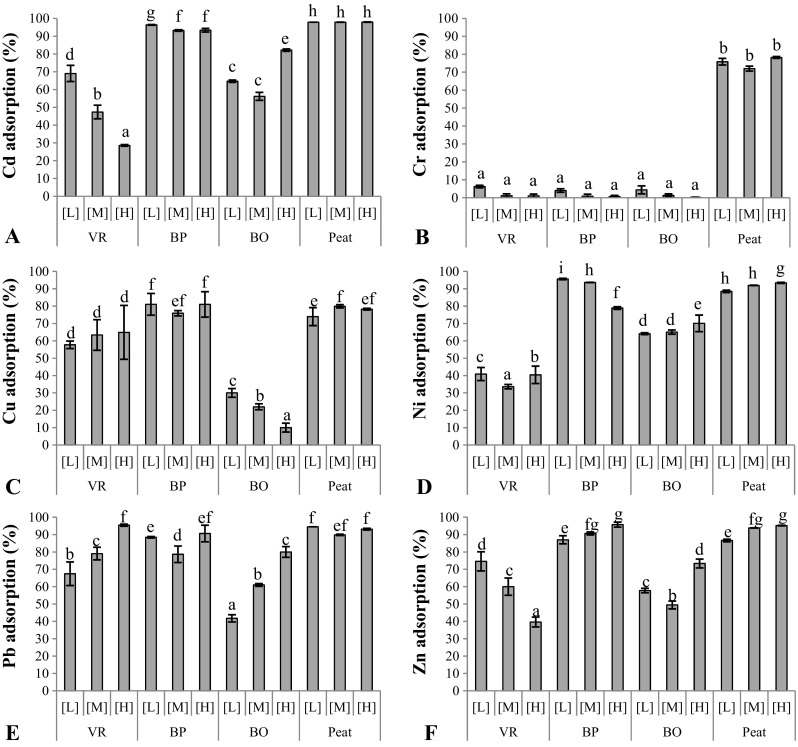
Capacity of individual materials for adsorption of heavy metals (**a**: Cd, **b**: Cr, **c**: Cu, **d**: Ni, **e**: Pb, **f**: Zn) at different concentration (high loading; [H]), diluted 1:1 (medium; [M]), or 1:10 (low; [L]). Different letters above bars indicate significant difference between means (*p* < 0.05, LSD test, *n* = 3)

For metal adsorption, most of the tested materials displayed a reduced capacity to adsorb at increasing metal concentrations, and the simultaneous presence of six metal ions likely led to competition for the same binding sites (Kalmykova et al. 2008, Trakal et al. 2011). Figure 2 shows that BP and peat performed best regarding adsorption of Cd, Cu, Ni, Pb, and Zn under static conditions. BO had a higher adsorption than VR for Cd and Ni, but lower absorption capacity for Pb and Cu. The adsorption performance of BO and VR varied the most as a function of solution concentrations, with BO having the highest absorption capacity at the highest metal load for four metals. Peat performed well in terms of Cr adsorption, whereas all the other materials adsorbed little Cr. Several studies demonstrate that Cr adsorption efficiency increases with decreasing pH (Pandey et al. 2010, Ajouyed et al. 2011), a likely explanation for the higher adsorption of Cr of peat.

### Dynamic retention capacity

Percolation through peat did not affect pH of the percolating solution, whereas the other materials and mixtures enhanced pH of the percolate. Metal-spiked solutions behaved similarly to pure water regarding changes in pH during percolation. Similarly, only peat maintained the initial high positive Eh of percolates (41.0 mV), while the other materials and mixtures reduced Eh. Low Eh may potentially affect Cr speciation, though reduced Eh and increasing pH act in opposite directions as to conserve initial Cr species, though on a longer term, Cr may be reduced in such a system (Kotaś and Stasicka 2000). No significant variation in Eh was observed between water used for conditioning columns and metal-spiked solutions used for the three percolations. Results related to pH and Eh variations within treatments after conditioning and during the percolations are presented in Fig. S1.

All materials and mixtures showed high retention of Phe, removing more than 94%, even after the third percolation event at the 21st day (Table 2). The VR/BO mixture had a lasting retention efficiency trend, probably due to a complementary in composition of hydrophobic binding sites. VR is not likely to affect the surface functional groups of biochar or clog its pores, due to its lack of soluble organic constituents. The peat/BO mixture removed relatively little of the Phe, possibly due to the interaction of the two materials which could mutually block or reduce the availability of adsorption sites.

**Table 2:** Phenanthrene and metal removal capacity (%) of materials and mixtures during three repeated (7, 14, 21 days) percolations. Material abbreviations as in Table 1. Within each percolation event, significant difference between treatments for individual compounds are indicated by different letters (*p* < 0.05, LSD test, *n* = 3, means ± SD)

	Time	VR		VR/BP		VR/BO		Peat		Peat/BP		Peat/BO	
	(days)												
	7	95 ± 1.1	d	98 ± 1.0	abc	99 ± 0.8	a	99 ± 0.6	ab	99 ± 0.5	b	96 ± 2.0	c
Phe	14	99 ± 0.1	k	98 ± 0.5	l	98 ± 0.7	l	99 ± 0.4	k	99 ± 0.2	k	99 ± 0.3	k
	21	94 ± 1.6	y	95 ± 1.5	xy	99 ± 0.0	w	98 ± 1.1	w	96 ± 1.3	x	98 ± 0.9	wx
	7	100 ± 0.0		100 ± 0.0		100 ± 0.0		100 ± 0.0		100 ± 0.0		100 ± 0.0	
Cd	14	100 ± 0.2		100 ± 0.0		100 ± 0.0		100 ± 0.0		100 ± 0.0		100 ± 0.0	
	21	100 ± 0.2		100 ± 0.1		100 ± 0.0		100 ± 0.1		100 ± 0.0		100 ± 0.0	
	7	80 ± 4.1	c	82 ± 2.8	c	69 ± 5.2	d	99 ± 0.5	a	91 ± 2.8	b	89 ± 3.9	b
Cr	14	57 ± 6.7	m	56 ± 0.5	m	44 ± 5.1	n	100 ± 0.5	k	90 ± 3.3	l	88 ± 5.9	l
	21	40 ± 3.0	y	36 ± 2.4	y	29 ± 6.6	z	98 ± 1.5	w	90 ± 3.2	x	85 ± 5.2	x
	7	97 ± 0.1	c	98 ± 0.2	b	99 ± 0.3	a	99 ± 0.6	a	99 ± 0.3	a	99 ± 0.1	a
Cu	14	98 ± 0.4	l	99 ± 0.2	k	100 ± 0.1	k	99 ± 0.3	k	99 ± 0.2	k	99 ± 0.1	k
	21	99 ± 0.1	x	100 ± 0.1	w	98 ± 0.5	y	99 ± 0.1	x	100 ± 0.1	w	100 ± 0.1	w
	7	98 ± 0.1	c	97 ± 0.2	c	98 ± 0.7	b	99 ± 0.2	a	99 ± 0.4	ab	99 ± 0.8	ab
Ni	14	98 ± 0.6	m	98 ± 0.5	m	99 ± 0.3	l	100 ± 0.1	k	99 ± 0.8	l	99 ± 0.1	l
	21	98 ± 0.5	x	99 ± 0.3	w	99 ± 0.2	w	99 ± 0.3	w	99 ± 0.2	w	100 ± 0.0	w
	7	100 ± 0.1	a	99 ± 0.1	b	100 ± 0.2	a	98 ± 0.5	c	99 ± 0.1	b	98 ± 0.5	c
Pb	14	100 ± 0.0	k	99 ± 0.2	l	100 ± 0.1	k	98 ± 0.5	m	99 ± 0.2	l	99 ± 0.2	l
	21	100 ± 0.2	w	100 ± 0.2	w	100 ± 0.1	w	98 ± 0.5	y	99 ± 0.1	x	99 ± 0.1	x
	7	99 ± 0.1	b	99 ± 0.1	b	100 ± 0.1	a	96 ± 0.5	d	98 ± 0.2	c	98 ± 0.6	c
Zn	14	100 ± 0.1	k	99 ± 0.2	l	100 ± 0.1	k	98 ± 0.5	n	99 ± 0.2	m	99 ± 0.2	m
	21	100 ± 0.1	w	100 ± 0.2	w	100 ± 0.1	w	98 ± 0.5	y	99 ± 0.1	x	99 ± 0.1	x

At day 21, the different adsorption behaviors of peat versus peat/BP, and of VR versus VR/BP, clearly indicated the role of BP in increasing the retention capacity of the mixtures. Still, both the mixtures containing BP only resulted in short-term Phe retention, probably due to adsorption by relatively weak physical bonds. The surface of biochar is highly heterogeneous due to the co-existence of carbonized and non-carbonized parts, which provide several adsorption possibilities for organic pollutants (Chen et al. 2008; Cao et al. 2009; Zheng et al. 2010). Moreover, biochar is characterized by an extended micro-porosity and a high surface area, properties specifically related to the adsorption of PAHs (Yu et al. 2009, Yang et al. 2010, Lou et al. 2011). Generally speaking, biochar obtained by low-temperature processes are typically characterized by higher surface area, increased micro-pore distribution, and a higher non-carbonized fraction compared to biochar produced at higher temperatures (Novotny et al. 2015). The superior performance of BO for removal of Phe after repeated infiltration may be explained by this difference in production (Uchimiya et al. 2010, Yang et al. 2010, Ahmad et al. 2012, Chen et al. 2008).

All material mixtures performed well regarding retention of Cd from percolating water (Table 2), and no significant differences were observed between treatments. For Cr adsorption, peat (alone or in mixtures) had the highest retention capacity and a stable performance over the three percolation events. No significant differences were observed between the other material mixtures over 21 days. According to Balan et al. (2009), lignin, cellulose, and hemicelluloses have crucial roles in sorption-coupled reduction removal processes. Moreover, several studies argue that biochar removes Cr via a preliminary electrostatic attraction, reduction to Cr(III), and subsequent complexation via O-containing functional groups (Dong et al. 2011, Choppala et al. 2012, and Bolan et al. 2013). These two mechanisms were probably active and persisted in peat/BP and peat/BO mixtures and were enhanced by the presence of peat.

Volcanic rock had a very low capacity for retention of Cu compared to the other materials, but mixing VR and biochar improved the retention capacity. Compared to other divalent metals, Cu has the highest affinity for carboxylic functional groups (Tong et al. 2011). Lima et al. (2010) reported that metal adsorption was higher for washed biochar than for untreated biochar due to an improved accessibility of their functional groups. Based on the features reported by these two studies, the enhanced Cu retention could be explained as an additive effect of these interactions. Specifically, the preferential formation of surface complexes between Cu and biochar functional groups can lead to exchange phenomena with other divalent metals previously adsorbed onto the mixtures, which may increase with time.

Nickel adsorption onto solid materials is most efficient in a pH range between 8 and 9 (Bartczak et al. 2015, Zhang and Wang 2015). Still, as shown in Table 2, peat retained almost all Ni entering the system. Moreover, peat had the highest and most persisting Ni removal capacity of the materials tested. The high retention capacity of peat/BO and peat/BP, and the increasing retention of VR-treatments, shows additive effects, possibly through increasing access to functional groups or binding sites.

For Pb, VR treatments had higher retention capacity than peat-containing mixtures. Malakootian et al. (2009) report that VR is capable of retaining large amounts of Pb due to its alkaline pH. This can also explain the increased retention capacity of Pb observed for peat when mixed with biochar. Lu et al. (2012) showed that Pb adsorption on biochar mainly occurs by outer-sphere exchange phenomena with other cations (Ca, K, Mg) that are commonly available on biochar surface. Moreover, for VR/BO and VR/BP, ion exchange mechanisms can easily have occurred due to the weak retention of VR compared to peat.

Table 2 shows Zn removal capacities, where VR adsorbed more Zn as compared to peat. Generally, adsorption processes occur through joint chemical and physical mechanisms (Tan et al. 2015). Sen and Khoo (2013) reported that the adsorption of Zn on clay surface occurs in two steps: a rapid adsorption of metal ions to the external surface, followed by possible slow diffusion towards the inner surfaces of the pores. The biochar samples used in this study showed both chemical and physical adsorption mechanisms that would contribute to the latter adsorption mechanism leading to high Zn removal in mixtures.

## Conclusion

A good green roof material must be light, highly porous, and resistant to water erosion. It should not be prone to clogging and provide an appropriate water retention/release performance. Both VR, peat, and their mixtures with biochar perform well regarding density and through the flow of water. For the retention of environmental pollutants, green roof materials should also allow a high mass transfer and be selective for pollutants of high concern. Here, the four materials used performed differently for different pollutants, and mixtures were clearly superior when the combined retention of all the tested pollutants are considered. This, along with the additional environmental benefits from using biochar from local and recycled materials, makes mixed green roof substrates containing biochar promising alternatives to present day’s use of less sustainable materials. The additional possibility to grow plants that support intensive use is another advantage of biochar mixtures (Cao et al. 2014, Nemati et al. 2014). The mixtures generally show improved retention behavior compared to pure materials. Their inclusion in roofs constructed with traditional roof materials will thus improve filtering performances, both quantitatively and qualitatively. Moreover, our study suggests that roof material mixtures can be designed using different types of biochar to facilitate retention of specific contaminants that cause problems locally.

## Electronic supplementary material

ESM 1 (DOCX 30 kb)
