# Supplementary material for: Assessment of addition of biochar to filtering mixtures for potential water pollutant removal
Source: Environ Sci Pollut Res Int. 2017 Nov 7;25(3):2167–74. doi: 10.1007/s11356-017-0650-6 (PMC5773637; doi:10.1007/s11356-017-0650-6)
Supplement: Supplementary file 1 — (DOCX 30 kb) [file 11356_2017_650_MOESM1_ESM.docx]

Supplementary Information

**Assessment of addition of biochar to filtering mixtures for potential water pollutant removal**

Lea Piscitelli†‡, Pierre-Adrien Rivier‡, Donato Mondelli§, Teodoro Miano§, Erik J. Joner*‡

† Mediterranean Agronomic Institute of Bari, Via Ceglie, 9, 70010 Valenzano-Bari, Italy

§ Department of Food, Plants and Soil Science, University of Bari Aldo Moro, Bari, Italy

‡ Division of Environment and Natural Resources, Norwegian Institute of Bioeconomy Research, Høyskoleveien 7, NO 1431 Ås, Norway

*Corresponding author e-mail: [erik.joner@nibio.no](mailto:erik.joner@nibio.no)

Ref.: **Ms. No. ESPR-D-17-00783**

Table S1. Characteristics of BO and BP (BP: Biochar from wood, BO; Biochar from olive husks).

|  | Total Carbon | Total Organic Carbon | Total Nitrogen | Dry matter |
| --- | --- | --- | --- | --- |
|  | % | | | |
| BP | 81 | 67.9 | < 0.08 | 74.71 |
| BO | 77.5 | 77 | < 0.9 | 98 |

Table S2. Heavy metals and Phenanthrene contents in blank samples containing only water and individual materials, measured in the bench experiment. Detection limits are indicated in brackets.

|  | Cd µg/l (1) | Cr µg/l (1) | Cu mg/l (0.001) | Ni  µg/l (0.9) | Pb µg/l (2) | Zn mg/l (0.004) | Phe mg/l |
| --- | --- | --- | --- | --- | --- | --- | --- |
| VR | < 1.000 | < 1.000 | 0.028 | 5.633 | 3.667 | 0.010 | 0.001 |
| BP | < 1.000 | < 1.000 | 0.028 | < 0.900 | 6.000 | 0.010 | 0.002 |
| BO | < 1.000 | < 1.000 | 0.034 | < 0.900 | 3.333 | 0.008 | 0.001 |
| Peat | < 1.000 | < 1.000 | 0.026 | 0.933 | 3.000 | 0.026 | 0.001 |
| Water | < 1.000 | < 1.000 | 0.019 | < 0.900 | < 2.000 | < 0.004 | 0.001 |

Figure S1. Red/ox potentials (mV) and pH of solutions after contact with materials and mixtures following the conditioning procedure with water (bars in blue) and after repeated percolations at 7 (in red), 14 (in green) and 21 (in violet) days.

Peat/BP

Peat/BO

Peat

VR

VR/BP

VR/BO
